# In Pursuit of Vitamin D in Plants

**DOI:** 10.3390/nu9020136

**Published:** 2017-02-13

**Authors:** Lucinda J. Black, Robyn M. Lucas, Jill L. Sherriff, Lars Olof Björn, Janet F. Bornman

**Affiliations:** 1 School of Public Health, Curtin University, Bentley 6102, Australia; j.sherriff@curtin.edu.au; 2 National Centre for Epidemiology and Population Health, Research School of Population Health, The Australian National University, Canberra 0200, Australia; robyn.lucas@anu.edu.au; 3 Department of Biology, Lund University, SE-223 62 Lund, Sweden; lars_olof.bjorn@biol.lu.se; 4 International Institute of Agri-Food Security (IIAFS), Curtin University, Bentley 6102, Australia; janet.bornman@curtin.edu.au

**Keywords:** vitamin D, 25-hydroxyvitamin D, 1,25-dihydroxyvitamin D, plants

## Abstract

Vitamin D deficiency is a global concern. Much research has concentrated on the endogenous synthesis of vitamin D in human skin following exposure to ultraviolet-B radiation (UV-B, 280–315 nm). In many regions of the world there is insufficient UV-B radiation during winter months for adequate vitamin D production, and even when there is sufficient UV-B radiation, lifestyles and concerns about the risks of sun exposure may lead to insufficient exposure and to vitamin D deficiency. In these situations, dietary intake of vitamin D from foods or supplements is important for maintaining optimal vitamin D status. Some foods, such as fatty fish and fish liver oils, certain meats, eggs, mushrooms, dairy, and fortified foods, can provide significant amounts of vitamin D when considered cumulatively across the diet. However, little research has focussed on assessing edible plant foods for potential vitamin D content. The biosynthesis of vitamin D in animals, fungi and yeasts is well established; it is less well known that vitamin D is also biosynthesised in plants. Research dates back to the early 1900s, beginning with in vivo experiments showing the anti-rachitic activity of plants consumed by animals with induced rickets, and in vitro experiments using analytical methods with limited sensitivity. The most sensitive, specific and reliable method for measuring vitamin D and its metabolites is by liquid chromatography tandem mass spectrometry (LC-MS/MS). These assays have only recently been customised to allow measurement in foods, including plant materials. This commentary focuses on the current knowledge and research gaps around vitamin D in plants, and the potential of edible plants as an additional source of vitamin D for humans.

## 1. Introduction

In most regions of the world, cutaneous synthesis following ultraviolet-B (UV-B, 280–315 nm) irradiation of 7-dehydrocholesterol in skin epidermal cells is the primary source of vitamin D for humans. When exposure to UV-B radiation is limited, because the ambient levels of UV-B radiation are low or skin is not exposed to the sun, dietary intake of vitamin D from food or supplements is required to maintain adequate vitamin D status. With the exception of mushrooms, the recognised natural dietary sources of vitamin D are animal-based (fish, meat, eggs and dairy), which raises concerns about low vitamin D intakes in populations that avoid, or consume low amounts of, animal products. For example, vitamin D deficiency is highly prevalent in India [1], where consumption of meat and dairy is low. A contribution of plant foods to dietary vitamin D intake could be important for such populations.

Liquid chromatography tandem mass spectrometry (LC-MS/MS) methods have only recently been applied to detect low concentrations of vitamin D and its metabolites in plants. These methods are superior to the previous in vivo and in vitro bioassays, which cannot distinguish between vitamin D_2_, vitamin D_3_ and their hydroxylated forms. LC-MS/MS methods have been used to measure vitamin D in some animal products [2] and a small number of plants [3,4,5]. Although algae are not vascular plants, and so fall outside the scope of this commentary, a macroalga, *Sargassum*, has been shown to have anti-rachitic activity [6], and both vitamin D_2_ and vitamin D_3_ have been found in fresh water phytoplankton [7].

**Figure 1:**
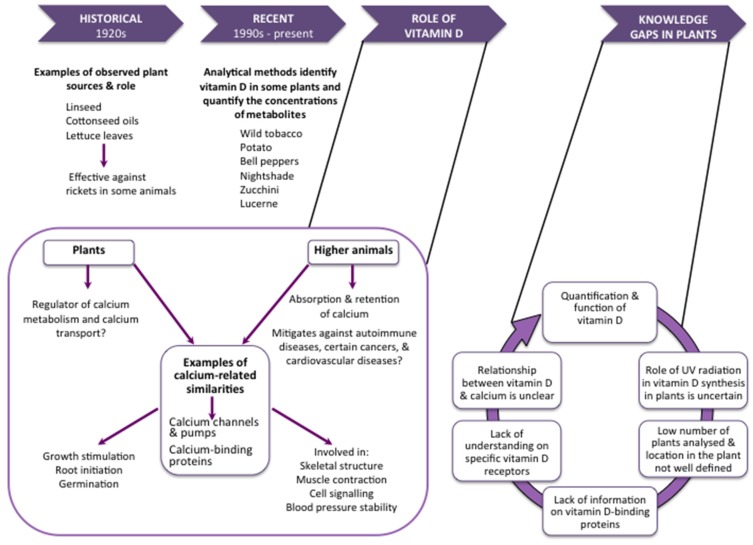
Vitamin D in plants: current knowledge and research gaps.

This commentary is an update on previous reviews of vitamin D in plants [5,8], and introduces several additional concepts: the lack of identification in plants of relevant proteins involved in vitamin D metabolism and transport; evidence suggesting that exposure to UV-B radiation may not be required for synthesis of vitamin D in plants; and the possibility of native Australian plants as a potential source of vitamin D. We briefly outline the current knowledge and research gaps around vitamin D in plants (Figure 1) and emphasise the potential role of vitamin D in plants for supplementing intake in humans.

## 2. Metabolism of Vitamin D in Plants

UV-B irradiation of sterol precursors results in the production of vitamin D_3_ in mammalian skin and vitamin D_2_ in yeasts and fungi. Both forms have been used as vitamin D supplements and in food fortification. Vitamin D_4_ (22-dihydroergocalciferol) has also been identified in fungi [9,10]. While mammalian and fungal cells each contain only one major sterol (7-dehydrocholesterol and ergosterol, respectively), plants have a complex sterol mixture, including sitosterol, stigmasterol, ergosterol, 7-dehydrocholesterol, campesterol and 24-methylcholesterol [11]. Cholesterol is only a minor sterol (1%–2% of total plant sterols) in most plant species, but can represent at least 10% of total sterols in some plants, such as the Solanaceae [12], a family of flowering plants including tomato, potato, eggplant, capsicum and tobacco. Plasma membranes from leaf cells of *Lycopersicon esculentum* (tomato plant) undergo changes in UV-induced absorbance with a trough at about 295 nm [11], pointing to the disappearance of a substance with an absorption peak at this wavelength. The wavelength at 295 nm is close to the action peak for previtamin D photosynthesis, which is thought to be due to the conversion of provitamin D which has an absorption maximum at this wavelength.

In humans, vitamin D_3_ synthesised in the epidermis is taken up into the bloodstream tightly bound to a vitamin D-binding protein. Both vitamin D_3_ (deriving from sun exposure and dietary sources) and vitamin D_2_ (deriving only from dietary sources) are metabolised in the liver through an hydroxylation pathway to the intermediate compound, 25-hydroxyvitamin D (25(OH)D), the major circulating and storage form. A further hydroxylation pathway, mainly in the kidney but also in other tissues, produces the active form of vitamin D, 1,25-dihydroxyvitamin D (1,25(OH)_2_D). The effects of 1,25(OH)_2_D in humans are mediated through ligation with nuclear vitamin D receptors (VDR) [13] and via membrane rapid-response receptors [14,15], both of which are found in most human tissues.

Neither VDR nor vitamin D-binding protein have yet been found in plants [16], but a VDR-like binding protein for 1,25(OH)_2_D_3_ has been described in *Solanum glaucophyllum* (waxy leaf nightshade), suggesting that similar mechanisms of action may be present in plants [17]. The enzymes involved in the two hydroxylation reactions (25-hydroxylase and 25-hydroxyvitamin D 1α-hydroxylase), along with 25(OH)D_3_ and 1,25(OH)_2_D_3_, in both free and glycosidic forms, have been identified in the leaves of *Solanum malacoxylon* [18] and *Cestrum diurnum* [19], plants known to be responsible for calcinosis in animals. Vitamin D_3_ is present in plants not only in free form, but also as a glycoside. Although it is not clear whether the glycoside and the free form of vitamin D_3_ are equally absorbed, glycosylation does not appear to substantially reduce the activity of vitamin D_3_: a study in chickens demonstrated that the vitamin D_3_ glycoside has an activity of 90%–95% compared with the aglycone [20].

## 3. The Function of Vitamin D in Plants

There may be some similarities between plants and animals in the way in which calcium and vitamin D are associated in regulatory processes. Vitamin D has a critical role in calcium and phosphate homeostasis in animals [16]. When blood calcium concentrations fall, there is upregulation of 1α-hydroxylase to increase 1,25(OH)_2_D concentrations. This results in increased intestinal absorption of calcium, along with decreased renal excretion, to restore normal blood calcium concentrations. When this mechanism is insufficient, bone metabolism is upregulated to release calcium from skeletal stores [21]. Plants have similar calcium channels and pumps to those found in animals [22], and calcium ions are a core regulator of plant cell physiology [16]. Calcium is required for stimulation of growth, root initiation and promotion of germination in plants [22]. 1,25(OH)_2_D_3_ has been shown to influence growth and calcium transport in roots of *Phaseolus vulgaris* (common bean) by increasing synthesis of calmodulin [8], a calcium-binding messenger protein found in all eukaryotic cells.

## 4. Vitamin D_2_ Content of Plants

Many plants contain endophytic fungi, which have cell membranes containing ergosterol [23,24,25]. Thus, vitamin D_2_ has been found in plants as a result of photoconversion of ergosterol in these fungal contaminants. In 1924, Hess and Weinstock found that UV-irradiated linseed oils, cottonseed oils and lettuce leaves were effective anti-rachitic agents when fed to rats [26]. Other studies have extended this work to show benefits in cattle [27] and chickens [28] with induced rickets. These experiments provided an in vivo assay of the anti-rachitic activity of various plants. The active compound was later identified as vitamin D_2_ produced from fungal contamination of the plants.

Perennial ryegrass, a common grass in permanent pastures, contains both ergosterol and vitamin D_2_ as a result of fungal contamination [3], and low amounts of vitamin D_2_ have been found in milk, presumably originating from fungal contamination of grass and hay [29]. However, although symbiotic fungi may improve resistance to stress and insects in the host plant [30], some fungi have been implicated in crop spoilage and/or toxicity to animals [31]. From a human nutrition perspective, there is conflicting evidence on the relative bioavailability and bioeffectiveness of vitamin D_2_ versus vitamin D_3_ [32,33,34,35].

## 5. Vitamin D_3_ Content of Plants

Vitamin D_3_ and 25(OH)D_3_ have been found mainly in the Solanaceae family, with research focussed on the leaves, which are known to be poisonous in large amounts. It is currently not clear whether vitamin D_3_ and its metabolites are present in the edible fruits of the Solanaceae. An extract from the leaves of the tomato plant was shown to significantly increase serum calcium concentrations in vitamin D-deficient rats, while the fruit was devoid of vitamin D-like activity [36]. To our knowledge, LC-MS/MS methods have not been used for analysing vitamin D_3_ and its metabolites in other edible fruits.

The presence of vitamin D_3_ may not be dependent on exposure to light in all plants. Previous studies in *Solanum glaucophyllum* have shown that vitamin D_3_ compounds, including 7-dehydrocholesterol, vitamin D_3_, 25(OH)D_3_ and 1,25(OH)_2_D_3_, are present in cultures grown in the absence of light [37,38]. In samples grown in vitro in darkness, both 25(OH)D_3_ and 1,25(OH)_2_D_3_ were present in the stem, leaf and (inedible) fruit [39]. Nevertheless, UV irradiation of the plants substantially increased concentrations of 1,25(OH)_2_D_3_ in the leaves. This suggests that treatment with UV radiation may be an effective method for increasing the content of vitamin D_3_ and its metabolites in plants (at least in the leaves), similar to the effect that UV irradiation has on increasing vitamin D_2_ in mushrooms [40].

Recently, Jäpelt and colleagues compared vitamin D_3_ and its hydroxylated metabolites in the UV-treated and untreated leaves of the tomato plant, waxy leaf nightshade and bell pepper [41]. Using LC-MS/MS methods, vitamin D_3_ was identified in the leaves of all three plants following treatment with UV radiation. Quantifiable 25(OH)D_3_ was detected in the UV-treated leaves of waxy leaf nightshade, tomato plant, and bell pepper, and was also in the untreated leaves of waxy leaf nightshade. Table 1 shows the concentrations of 7-dehydrocholesterol, vitamin D_3_, 25(OH)D_3_ and 1,25(OH)_2_D_3_, measured by LC-MS/MS methods, in the leaves of various plant species with and without treatment with UV irradiation.

## 6. Research Gaps

**Figure 2:**
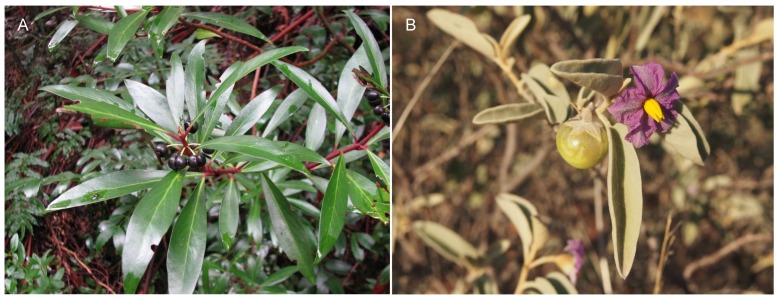
(**A**) *Tasmannia lanceolata* (Tasmanian pepper) (Credit: Mark Marathon—Own work, CC BY-SA 3.0, https://commons.wikimedia.org/w/index.php?curid=28133459); (**B**) *Solanum centrale* (bush tomato) (Credit: Melburnian—Self-photographed, CC BY 3.0, https://commons.wikimedia.org/w/index.php?curid=10145927).

There are relatively few studies quantifying vitamin D_3_ and its metabolites in plants, and even fewer investigating plant parts other than leaves. Furthermore, the chemical configuration and availability of different vitamin D_3_ metabolites in plants remain unknown. There is also currently little information on the occurrence and concentration of vitamin D_3_ in plants, or the vitamin D_3_ pathway, including binding proteins, receptors and activating enzymes. Treatment of edible plants or plant parts with UV radiation may be an approach for increasing vitamin D in the food supply, particularly for those who avoid animal products. However, with respect to the Solanaceae family, the toxicity of the leaves prohibits their use as a potential source of vitamin D. Further exploration of vitamin D_3_ in native Australian plant foods may be warranted, since they grow naturally in high UV radiation environments and many have medicinal properties. Examples of native plant foods include *Tasmannia lanceolata* (Tasmanian pepper), which has a long history of use by Australian Aboriginal people as a food flavouring and is a good source of calcium; and *Solanum centrale* (bush tomato), belonging to the Solanaceae family (Figure 2). A major challenge will be to ensure that methods to analyse vitamin D_3_ and its metabolites are reliable and accurate when measuring low concentrations in the complex matrices presented by different plant parts. 

## Figures and Tables

**Table 1:** Concentrations of vitamin D_3_ metabolites (dry weight) in plant leaves from the Solanaceae family, measured by liquid chromatography tandem mass spectrometry.

Species	7-dehydrocholesterol (μg/g)	Vitamin D_3_ (μg/g)	25(OH)D_3_ (μg/g)	1,25(OH)_2_D_3_ (μg/g)
**Non-irradiated**				
*Lycopersicon esculentum* (tomato plant)	0.47 [4] ^1^	Not identified [4] ^1^ 0.0017 [41] ^2^	<0.00002 [41] ^2^	<0.0001 [41] ^2^
*Solanum glaucophyllum* (waxy leaf nightshade)	0.67 [4] ^1^	Not identified [4] ^1^ 0.0032 [41] ^2^	0.0008 [41] ^2^	<0.0001 [41] ^2^
*Capsicum annuum* (bell pepper)	0.03 [4] ^1^	Not identified [4] ^1^ <0.00002 [41] ^2^	<0.00002 [41] ^2^	<0.0001 [41] ^2^
**UV-irradiated**				
*Lycopersicon esculentum* (tomato plant)	0.23 [4] ^1^	0.09 [4] ^1^ 0.1 [41] ^2^	0.0043 [41] ^2^	<0.0001 [41] ^2^
*Solanum glaucophyllum* (waxy leaf nightshade)	1.26 [4] ^1^	0.21 [4] ^1^ 0.2 [41] ^2^	0.031 [41] ^2^	0.032 [41] ^2^
*Capsicum annuum* (bell pepper)	0.03 [4] ^1^	Not identified [4] ^1^ 0.0029 [41] ^2^	0.0005 [41] ^2^	<0.0001 [41] ^2^

^1^ Atmospheric pressure chemical ionisation liquid chromatography tandem mass spectrometry; ^2^ Liquid chromatography-electrospray ionisation tandem mass spectrometry.

## Abbreviations

The following abbreviations are used in this manuscript:

UV-B	Ultraviolet-B radiation
25(OH)D	25-hydroxyvitamin D
1,25(OH)_2_D	1,25-dihydroxyvitamin D
VDR	Vitamin D receptor
LC-MS/MS	Liquid-chromatography tandem mass spectrometry
